# Men’s and women’s exposure and perpetration of partner violence: an epidemiological study from Sweden

**DOI:** 10.1186/1471-2458-12-945

**Published:** 2012-11-02

**Authors:** Solveig Lövestad, Gunilla Krantz

**Affiliations:** 1 Department of Community Medicine and Public Health/Social Medicine, The Sahlgrenska Academy at University of Gothenburg, Göteborg, Box 453, 40530, Sweden

**Keywords:** Intimate partner violence, Controlling behaviour, Risk factors, Men, Women, Sweden

## Abstract

**Background:**

Over the past 30 years, intimate partner violence (IPV) against women and its health consequences has become a well established research area and is recognized worldwide as a significant public health issue. Studies on IPV directed at men are less explored, however recently women’s use of IPV and men’s victimization is gaining growing attention. Earlier population-based studies performed in Sweden have primarily investigated men’s violence against women, while women’s use of violence and men’s exposure as well as the existence of controlling behaviours have been neglected research areas This explorative study investigated the exposure to and perpetration of intimate partner violence, the use of control behaviours and the associated risk factors among a sample of Swedish men and women.

**Methods:**

This cross-sectional population-based study included 173 men and 251 women of age 18–65 randomly selected among the Swedish population. A questionnaire based on the revised Conflicts Tactics Scale (CTS2) and the subscale ‘isolating control’ from the Controlling Behaviour Scale (CBS) was used to collect data on violence exposure and perpetration. Regression analyses were used for risk factor assessment.

**Results:**

More men (11%) than women (8%) reported exposure to physical assault in the past year, while more women reported exposure to sexual coercion. Duration of present relationship ≤ 3 years was identified as a significant risk factor for men’s exposure. Young age, lack of social support and being single, constituted risk factors for women’s exposure. Surprisingly many men (37%) and women (41%) also reported exposure to controlling behaviours.

**Conclusions:**

In partner violence research, both men’s and women’s exposure should be explored however findings need to be interpreted with caution. This first study in a Swedish sample establishes the basis for future investigations on partner violence and coercive control tactics.

## Background

Over the past 30 years, intimate partner violence (IPV) against women and its health consequences has become a well established research area and is recognized worldwide as a significant public health issue [1,2]. Studies on IPV directed at men are less explored, however recently women′s use of IPV and men’s victimization is gaining growing attention [3,4].

The past few decades have featured an ongoing debate over between two distinctly different standpoints within IPV research: the ‘family violence perspective’ and the ‘feminist perspective’ [5]. Researchers stemming from the ‘family violence perspective’ claim gender symmetry in violence; i.e., both men and women use violence within relationships [6,7]. Alternatively, ‘feminist perspective’ researchers claim that men are the main perpetrators of violence and women mainly use violence in self-defence or to protect someone they care for (gender asymmetry) [3,8,9]. Even though women report the use of physical violence, sometimes to the same extent as men, studies indicate that men display more aggressive and sexually coercive behaviour than women, and that women are more likely to be injured than men [4,6,8,10-13].

Johnson [5], Stark [14] and Graham-Kevan [15] have suggested that it is crucial to check for the existence of coercive control tactics in order to understand IPV and subsequently intervene in an effective manner. Coercive control tactics have different causes as well as consequences, and require other types of interventions compared to violence without control tactics [5].

Earlier population-based studies performed in Sweden have primarily investigated men’s violence against women, while women’s use of violence and men’s exposure as well as the existence of controlling behaviours have been neglected research areas [16].

The aim of this explorative study was therefore to investigate, in a sample of Swedish men and women, both exposure to and perpetration of intimate partner violence, including controlling behaviours and the associated socio-demographic and psychosocial risk factors.

## Methods

### Design and population

The data collection for this population-based, cross-sectional study took place in January 2009. A questionnaire was sent to a randomly selected sample of 505 men and 502 women, aged 18–65 by Statistics Sweden. The questionnaire contained items on violent acts, controlling behaviours and additional socio-demographic and psychosocial variables.

The response rate was 56.5% (*n* = 282) for women and 43.5% for men (*n* = 217), and for the total sample it was 49.6% (*n* = 499). Of the non-respondents, the majority were unmarried compared to the respondents (54.7% vs. 40.1%); further 12.8% were divorced and 20.3% were born in a foreign country while corresponding figures for the respondents were 10.4% and 13.4%, respectively.

A further 75 individuals had to be excluded as they did not respond to any of the violence behaviour questions. The final sample consisted of 424 individuals, 173 (40.8%) men and 251 (59.2%) women. The age range was 19 to 65, with an average age of 44.3 (*SD* = 13.53) for men and 42.8 (*SD* = 13.59) for women.

### The questionnaire

#### Dependent variables

The questionnaire contained the revised version of the Conflict Tactics Scales (CTS2) constructed by Straus et al. [17] to estimate physical and sexual violence occurrence and the subscale ‘isolating control’ (five items) from the Controlling Behaviour Scale (CBS) developed by Graham-Kevan and Archer [15].

From the CTS2 we included the Physical Assault subscale (PA) (physical violent acts like slapping or kicking, see Table 1 for items) and the Sexual Coercion subscale (SC) (behaviour that intends to force the partner, either verbally or physically, to engage in sexual activity, items displayed in Table 1) [17]. The CTS2 measures concrete acts and events of violence between intimate partners even though only one person in the couple is asked [17-19]. It has been extensively used within violence research and its validity and reliability are considered high [17].

**Table 1:** Exposure to violence presented as prevalence

**Exposure to physical assault and sexual coercion (“Partner did this to me”)**				
**Forms of violence**	**Men n = 173**		**Women n = 251**	
	**Past year%(n)**	**Earlier in life%(n)**	**Past Year%(n)**	**Earlier in life%(n)**
**Physical assault**				
Threw something	4.0 (7)	5.2 (9)	1.6 (4)	7.6 (19)
Twisted arm or hair	1.7 (3)	1.7 (3)	1.6 (4)	6.0 (15)
Pushed or shoved	7.5 (13)	5.8 (10)	5.6 (14)	10.8 (27)
Used knife or tool	0.0 (0)	4.0 (7)	0.8 (2)	4.4 (11)
Hit with something that could hurt	1.7 (3)	3.5 (6)	0.0 (0)	3.6 (9)
Choked	0.6 (1)	1.2 (2)	1.2 (3)	4.4 (11)
Slammed against a wall	0.0 (0)	0.6 (1)	1.2 (3)	6.8 (17)
Beat up	0.0 (0)	1.7 (3)	1.2 (3)	5.2 (13)
Grabbed	2.9 (5)	3.5 (6)	2.4 (6)	8.0 (20)
Slapped	4.6 (8)	5.8 (10)	0.8 (2)	6.8 (17)
Burned or scaled	0.0 (0)	0.6 (1)	0.0 (0)	2.8 (7)
Kicked	1.2 (2)	1.7 (3)	0.8 (2)	3.2 (8)
***Total exposed individuals***^***a***^	***11.0 (19)***	***11.0 (19)***	***8.0 (20)***	***15.9 (40)***
**Sexual coercion**				
Made partner have sex without condom	0.0 (0)	1.2 (2)	0.8 (2)	3.2 (8)
Used force to have sex	0.0 (0)	0.6 (1)	0.4 (1)	3.6 (9)
Insisted on having sex	0.6 (1)	2.9 (5)	2.8 (7)	8.0 (20)
Used threats to have sex	0.0 (0)	0.6 (1)	0.8 (2)	3.6 (9)
***Total exposed individuals***^***a***^	***0.6 (1)***	***3.5 (6)***	***3.2 (8)***	***9.6 (24)***

N = 424

^*a.*^*The number of individuals exposed to at least one of the acts.*

The randomly selected men and women responded to exactly the same items and were asked to indicate whether they had been exposed to or used any of the violent acts or controlling behaviours included. A five point scale was used to indicate the frequency of the various violent acts and controlling behaviours (from ‘not at all’ to ‘more than five times’) for the *past year*. For *earlier in life* exposure and perpetration, only ‘yes’ or ‘no’ alternatives were given without inquiring about frequency.

The prevalence was estimated for each of the acts and as a composite measurement for each of the subscales. Dichotomised variables were constructed for the two forms of violence from the CTS2. Exposure or perpetration was present if a person had been exposed to or perpetrated one or more acts at least once in the *past year* or ever over the time period *earlier in life*. Concerning the subscale of isolating control, exposure was present if a person had been exposed to one or more controlling behaviours at least once during their lifetime.

The internal consistency of the subscales from CTS2 for lifetime prevalence was the following: PA (12 items), alpha 0.88 for men and 0.94 for women; the SC subscale (4 items) showed alpha 0.73 for men and 0.82 for women. For the lifetime prevalence of isolating control (5 items), alpha was 0.77 for men and 0.85 for women.

#### Independent variables

Socio-demographic and psychosocial variables were as analyzed to determine whether these were independent risk factors. *Age* was dichotomised into ‘18-30’ versus ‘31-65’ years of age, using the older age group as the reference in the multivariate analysis. *Country of birth* was classified into ‘born in Sweden or any other Scandinavian country’ as opposed to being ‘born outside of Scandinavia.’ *Educational level* was dichotomised into ‘basic education’, with 13 years or less considered as the exposed, and ‘university education’ as the reference category.

*Civil status* was classified into categories ‘married/cohabitant’ versus ‘not married’ or ‘cohabitant’*. Occupational level* was divided into five groups then dichotomised with those in paid employment as the reference category. *Total household income* was defined as the total household income before tax and classified into three groups. It was dichotomised into ‘0-39 999 Swedish crowns (SEK) per month’ (equivalent to approx 6 400 USD per month) and with ‘> 40 000 SEK’ as the reference category. The variable *children living at home* was dichotomised as ‘not having children at home’ (reference) and ‘all other alternatives’ making up the exposed group. *Duration of present relationship* was dichotomized as ‘< 3 years’ as the exposure category and ‘≥ 3 years’ as the reference category. *Grown up in a home with violence* was defined as physical, mental or sexual violence between parents or other adults that the participant had grown up with. It was constructed as a ‘yes’ or ‘no’ variable with ‘no’ as the reference category. The measure *Having social support* has been used in a previous study from the Swedish Level of living surveys (LNU) [20] and was constructed out of four items inquiring about the following: support when ill; when in need of company; when in need of discussing personal matters; and in need of borrowing 15 000 SEK (approx. 2200 USD). From this, a dichotomised variable was constructed where a ‘no’ response to all items was categorised as poor social support and at least one ‘yes’ response was categorised as having good social support.

### Statistical methods

Prevalence, frequency and associations were calculated for violence perpetration and exposure.

For the bi-and multivariate analysis, due to the small sample size, acts of PA were merged with the SC acts (labelled as PA/SC). Further, the time-related variables ‘past year’ and ‘earlier in life’ were merged into ‘lifetime prevalence’. Bivariate and multivariate analyses were used to investigate the associations between independent (socio- demographic- and psychosocial variables) and dependent variables (PA/SC) by use of odds ratios (OR) with statistical significance determined at the 95% confidence interval level.

In the logistic regression analyses, variables statistically significant in the bivariate analyses were entered one-by-one in a stepwise fashion for causal chain relationship and confounding analysis. As no of these variables showed high inter-correlation, they were all used in the multivariate analysis.

The Statistical Package for the Social Sciences (SPSS) version 17.0 was used for all statistical analyses.

### Ethical considerations

The Regional Ethics Review Board in Gothenburg gave approval for this research project, reference number 527–08.

## Results

### Socio- demographic and psychosocial characteristics

The majority of men (59.5%) and women (50.6%) had only basic education with less than 13 years of schooling (Table 2). Most of the respondents were married, cohabiting or living in registered partnerships. More women than men (37.8% vs. 35.3%) reported having poor social support.

**Table 2:** Socio-demographic and psychosocial variables

**Variables**	**Total population**	**Men N =173**	**Women N=251**
	**% (n)**	**% (n)**	**% (n)**
**Age groups**			
18-40	41.0 (174)	42.8 (74)	39.8 (100)
41-65	59.0 (250)	57.2 (99)	60.2 (151)
**Country of birth**			
Sweden & other Scandinavian Countries	89.4 (379)	87.9 (152)	90.4 (227)
Other European Countries & outside of Europe	10.6 (45)	12.1 (21)	9.6 (24)
**Educational level**			
Basic Education ≤ 13 yrs	54.2 (230)	59.5 (103)	50.6 (127)
University Education	45.5 (193)	39.9 (69)	49.4 (124)
**Civil status**			
Married/reg. partnership/cohabitant	76.7 (325)	76.9 (133)	76.5 (192)
Boy- Girlfriend/Single/Divorced/Widowed	22.6 (96)	22.5 (39)	22.7 (57)
**Occupation**			
Employed	72.4 (307)	79.2 (137)	67.7 (170)
Unemployed/other/Parental leave	9.7 (41)	4.6 (8)	13.1 (33)
Student	7.1 (30)	5.2 (9)	8.4 (21)
Pensioner (include Early retirement & Sickness- Disability pension)	8.3 (35)	8.1 (14)	8.4 (21)
Sick leave ≥ 3 months	1.7 (7)	1.2 (2)	2.0 (5)
**Total household income per month (SEK)**			
0 -19 999	13.4 (57)	8.7 (15)	16.7 (42)
20 000 – 39 999	34.0 (144)	36.4 (63)	32.3 (81)
**≥** 40 000	50.5 (214)	53.2 (92)	48.6 (122)
**Children living at home**			
Yes	44.8 (190)	43.4 (75)	45.8 (115)
No	55.0 (233)	56.6 (98)	53.8 (135)
**Duration of present relationship**			
≤ 1 year – 3 years	16.7 (71)	17.3 (30)	16.3 (41)
4 - ≥ 10 years	70.8 (300)	69.4 (120)	71.7 (180)
**Grew up with violence in the home (physical/psychological/sexual)**			
No/Do not know	90.6 (384)	93.6 (162)	88.4 (222)
Yes	8.3 (35)	5.2 (9)	10.4 (26)
**Social network and support: Having a relative/friend willing to help when falling ill**			
Yes	93.2 (395)	91.3 (158)	94.4 (237)
No/do not know	5.7 (24)	6.9 (12)	4.8 (12)
**Having a relative/friend willing to help when in need of company**			
Yes	92.5 (392)	87.3 (151)	96.0 (241)
No/do not know	5.9 (25)	9.2 (16)	3.6 (9)
**Having a relative/friend willing to help when in personal worries**			
Yes	89.9 (381)	83.2 (144)	94.4 (237)
No/do not know	8.7 (37)	13.9 (24)	5.2 (13)
**Having a relative/friend willing to help when in need of a loan of 15 000 SEK (approx. 2200 USD)**			
Yes	65.6 (278)	68.2 (118)	63.7 (160)
No/do not know	32.5 (138)	28.9 (50)	35.1 (88)

N = 424.

### Violence exposure

We found that more men than women (11% vs. 8.0%) reported exposure to one or more acts of PA during *the past year* (Table 1). For the time period *earlier in life*, however, more women (15.9%) than men (11.0%) reported exposure to such acts. SC affected women to a higher extent than men since only one man reported exposure to such an act in *the past year* compared to eight women. For *earlier in life*, 9.6% of the women and 3.5% of the men reported exposure to such acts.

### Violence perpetration

Men reported perpetrating PA to the same magnitude for *past year* (8.1%) as for *earlier in life* (8.1%), whereas 5.2% of the women reported the use of PA towards their partner in *the past year* and 11.6% for *earlier in life* (Table 3). More men than women (5.2% vs. 0.8%) reported the use of SC against their partner in *the past year*.

**Table 3:** Violence perpetration presented as prevalence

**Perpetration of physical assault and sexual coercion (“I did this to partner”)**				
**Forms of violence**	**Men n = 173**		**Women n = 251**	
	**Past year%(n)**	**Earlier in life%(n)**	**Past Year%(n)**	**Earlier in life%(n)**
**Physical assault**				
Threw something	1.7 (3)	1.2 (2)	1.6 (4)	6.0 (15)
Twisted arm or hair	1.2 (2)	2.9 (5)	1.6 (4)	3.2 (8)
Pushed or shoved	5.2 (9)	5.2 (9)	2.8 (7)	6.4 (16)
Used knife or tool	0.0 (0)	1.2 (2)	0.0 (0)	2.0 (5)
Hit with something that could hurt	0.0 (0)	1.7 (3)	0.4 (1)	2.0 (5)
Choked	0.0 (0)	1.7 (3)	0.4 (1)	2.0 (5)
Slammed against a wall	0.0 (0)	1.7 (3)	0.0 (0)	2.4 (6)
Beat up	0.0 (0)	1.2 (2)	0.8 (2)	2.4 (6)
Grabbed	2.3 (4)	4.0 (7)	1.2 (3)	4.4 (11)
Slapped	0.6 (1)	2.3 (4)	0.8 (2)	4.4 (11)
Burned or scaled	0.0 (0)	0.6 (1)	0.0 (0)	2.0 (5)
Kicked	0.6 (1)	1.2 (2)	0.4 (1)	2.0 (5)
***Total individuals***^***a***^	***8.1 (14)***	***8.1 (14)***	***5.2 (13)***	***11.6 (29)***
**Sexual coercion**				
Made partner have sex without condom	0,0 (0)	1.2 (2)	0.4(1)	2.0 (5)
Used force to have sex	0,0 (0)	0.6 (1)	0.0 (0)	2.0 (5)
Insisted on having sex	5,2 (9)	1.7 (3)	0.4 (1)	2.0 (5)
Used threats to have sex	0,0 (0)	0.6 (1)	0.0 (0)	2.0 (5)
***Total individuals***^***a***^	***5.2 (9)***	***2.3 (4)***	***0.8 (2)***	***2.0 (5)***

N = 424.

^*a*^*The number of individuals that had used at least one of the acts.*

### Isolating control

Over the lifetime, women were more exposed to controlling behaviours than men, however a considerable proportion of the men also reported such exposure (41.4% and 37.0% respectively) (Table 4). Interestingly, for three of the items, men and women’s exposure were of similar size but for two of the items (item 1 and 3) women reported exposure to a considerably higher extent than men, both mirroring activities taking place outside of the partners’ presence. Women to a larger extent also reported scores on all five items (7.6% vs. 2.9%), which in our interpretation is a sign of being under severe control from the partner.

**Table 4:** Lifetime prevalence of isolating control by intimate partner

**Isolating control (“Partner did this to me”)**		
**Variables**	**Men: n = 173**% **(n)**	**Women: n = 251**% **(n)**
1. Tried to restrict time spent with family/friends	8.7 (15)	15.5 (39)
2. Wanted to know where the other went and who the other spoke to when not together	26.0 (45)	28.3 (71)
3. Tried to limit the others’ activities outside the relationship	9.8 (17)	16.7 (42)
4. Felt suspicious and jealous of the other	27.2 (47)	27.1 (68)
5. Tried to control the others activities	12.1 (21)	14.7 (37)
***Total exposed individuals***^***a***^	***37.0 (64)***	***41.4 (104)***
**Total scores on isolating control**		
1 score	12.1 (21)	15.9 (40)
2 scores	11.6 (20)	9.2 (23)
3 scores	7.5 (13)	4.8 (12)
4 scores	2.9 (5)	4.0 (10)
5 scores	2.9 (5)	7.6 (19)
Non responses	1.2 (2)	1.2 (3)
**Total**	**100 (173)**	**100 (251)**

N = 424.

^*a.*^*The number of individuals exposed to at least one of the acts.*

### Exposure and perpetration of physical assault/sexual coercion and isolating control

As a considerable proportion of the men were exposed to PA/SC, we also wanted to investigate whether these men also used any form of violence against their partner. We found that of those exposed, 63.9% (n=23) also reported perpetration of PA/SC in their lifetime. For women this relationship was different as 39.4% (n=26) of those exposed also used PA/SC towards their partner.

### Crude associations

For lifetime exposure to PA/SC among men, statistically significant risk factors were attributable to the younger age group (OR 3.19; 1.36 – 7.49), being single or unmarried (OR 3.57; 1.36 – 7.49), and having a relationship of less than three years duration (OR 4.85; 1.94 – 12.12) (Table 5). For women, the most prominent risk factor was to be single or unmarried (OR 4.30; 2.29 – 8.09) and being in a relationship of short duration (OR 2.60; 1.26 - 5.38). Unemployment, low household income and having poor social network and support also proved statistically significant for women (Table 5).

**Table 5:** Associations between socio- demographic and psychosocial factors and exposure to violence

**Exposure to physical assault & sexual coercion lifetime prevalence (Past year + Earlier in life)**								
**Socio – demographic variables**	**Men: N = 173**				**Women: N = 251**			
	**Tot. N**	**Tot. exposed%(n)**	**Crude OR (95%CI)**	**Adjusted OR (95%CI)**^**a**^	**Tot. N**	**Tot. exposed%(n)**	**Crude OR (95%CI)**	**Adjusted OR (95**% **CI)**^**b**^
**Age groups**								
31-65	142	66.7 (24)	1		186	61.8 (42)	1	
18-30	31	33.3 (12)	3.19 (1.36 – 7.49)	1.63 (0.43 - 6.21)	65	38.2 (26)	2.24 (1.22 – 4.10)	2.14 (0.83 – 5.49)
**Educational level**								
University Education	69	50.0 (18)	1		124	47.1 (32)	1	
Basic Education ≤ 13 yrs	103	50.0 (18)	0.60 (0.29 – 1.27)	-	127	52.9 (36)	1.18 (0.68 – 2.06)	-
**Civil status**								
Married/reg. partnership/cohabitant	133	58.3 (21)	1		192	56.7 (39)	1	
Boy-Girlfriend/Single/Divorced/Widowed	39	41.7 (15)	3.57 (1.60 – 7.10)	2.12 (0.51 – 8.75)	57	43.3 (29)	4.30 (2.29 – 8.09)	3.10 (1.06 – 9.12)
**Occupation**								
Employed	137	85.7 (30)	1		170	58.2 (39)	1	
Unemployed	33	14.3 (5)	0.62 (0.22 - 1.74)	-	80	41.8 (28)	1.82 (1.01 – 3.26)	-
**Total Household income per Month (SEK)**								
≥ 40 000	92	47.2 (17)	1		122	34.3 (23)	1	
0-39 999	78	52.8 (19)	1.41 (0.67 – 2.95)	0.62 (0.21 – 1.86)	123	65.7 (44)	2.44 (1.36 – 4.38)	1.40 (0.67 – 2.90)
**Duration of present relationship**								
4 - ≥ 10 years	120	55.6 (15)	1		180	68.6 (35)	1	
≤ 1 year – 3 years	30	44.4 (12)	4.85 (1.94 – 12.12)	4.20 (1.15 – 15.40)	41	31.4 (16)	2.60 (1.25 - 5.38)	1.14 (0.38 – 3.36)
**Having social network/support**								
Yes	107	61.1 (22)	1		152	47.7 (31)	1	
No/Do not know	61	38.9 (14)	1.13 (0.53 -2.42)	-	95	52.3 (34)	2.23 (1.25 – 3.98)	2.79 (1.31 – 5.92)

N = 424.

^a^*Adjusted for age group, civil status, household income and duration of present relationship*.

^*b*^*Adjusted for age, civil status, household income, duration of relationship and social support*.

Results from the regression analysis for violence exposure over the lifetime are displayed in Table 5. For men, a relationship of less than three years duration remained the only statistically significant risk factor for PA/SC (OR 4.20; 1.15 – 15.40) while poor social support (OR 2.79; 1.31 – 5.92) and being single or unmarried (OR 3.10; 1.06 – 9.12) remained statistically significant risk factors for women.

## Discussion

In this unique population based study investigating men’s and women’s violence exposure and perpetration we found that exposure to PA was slightly higher in men than in women in the past year while earlier in life estimates were higher for women than for men. Men’s exposure to SC was negligible while women’s exposure was considerable (3% in the past year). Both men and women reported use of physical violence against their partner, men to a higher extent than women, while sexual coercion was reported mainly by men (5%). We further looked at exposure and perpetration combined, and found that a considerable proportion of the men exposed to PA/SC also used such violence (64%), while this was less commonly seen in women. Furthermore, considerably more women indicated exposure to all the different controlling behaviours investigated.

The only identified risk factor for men’s violence exposure was short duration of present relationship. Women who were single, divorced or widowed were at an increased risk of physical/sexual assault as were those with a poor social network.

### Findings in relation to other studies

Regarding exposure to PA during the past year, there is no Swedish population-based study available that estimates men’s exposure, while our findings for women concur with earlier reports [21] However, a study from Straus and colleagues [17] based on a sample of students found that more men (49%) than women (31%) reported exposure to PA during past year which is similar to our findings although our estimates were considerably lower in size. We found however that earlier in life estimates of PA where higher for women than for men. We also noted that women reported exposure to acts considered to be “severe”; for example, being “slammed against the wall,” “grabbed,” “burned or scaled” and “choked” more often than men, which is consistent with previous findings indicating that men use serious assaults to a larger extent than women [14]. Since women mostly are physically disadvantaged [19] we hypothesize that women may feel more threatened than men and subsequently recall bias would be less in women than in men. Such a hypothesis may also explain why men’s reported exposure to physical assault for the period earlier in life was lower than expected (it was of the same size as for the past year) . Support for this reasoning is also found in a study by Dobash and Dobash [8] where men described women’s violence as “insignificant,” “comical” and “ludicrous” (pp 340), hereby demonstrating that the violence was less frightening to them and less import to recall.

Somewhat to our surprise, we found that more women than men reported perpetration of PA for the time period ‘earlier in life’. Other studies describe self-defence as an important motive for woman’s violence [9,22]. Due to gender norms, women’s use of violence is further seen as less acceptable and may evoke shame in women, therefore women may remember their own use of violence to a higher extent than men [23]. However, since this particular study did not explore the motives behind the acts it is difficult to draw any further conclusions.

Throughout our analyses, more women than men reported exposure to SC during the past year and earlier in life and this is consistent with earlier research [4,9,10,13]. It is widely known that women exposed to SC (with or without physical abuse) are more likely to suffer serious health problems compared to women physically abused but not exposed to any kind of SC [24,25]. This indicates the need for further efforts regarding both prevention and interventions.

More women than men were exposed to isolating control. According to earlier findings, women are mainly exposed to a combination of violent acts and coercive control tactics such as threats and intimidation while men are the prime perpetrators of such violence in heterosexual relationships [5,14,15]. Traditional gender norms, power structures and gender inequalities between men and women are core factors that give rise to coercive control tactics [14,26]. We did, however, also find that men reported exposure to isolating control which points to the existence of female perpetrators [5,15]. It may be that exposure to coercive control tactics differ in characteristics and long-term consequences for men and women and therefore future investigations, both qualitative and quantitative, should explore the dynamics and emotions surrounding coercive control tactics.

In summary, we found in this study that both men and women are exposed but also use physical violence and controlling behaviours within their relationship, thus suggesting gender symmetry. Such findings have been published previously, mainly in the USA and other Anglo-Saxon countries [15,17]. However, as the motives were not further explored, we are not able to state with accuracy the reason for women’s use of violence, i.e. in self-defence or also in aggression.

### Associations with socio-demographic and psychosocial factors and violence

Similar to other studies of women, poor social support [27] and belonging to the younger age group were significant risk factors for exposure to violence [28,29].

Good social support is commonly found to be a protective factor against IPV and against the recurrence of IPV for women exposed earlier in life [27] and this is also what we found in this study. Social support contributes to making women feel valued, enhances their self-esteem and functions as a practical resource to assist when exposed to violence [27,30,31]. On the other hand, being young and exposed to poor social support signals being in a vulnerable position that may hamper women’s help-seeking.

For men, due to the small sample size, we found that short duration of present relationship was the only factor that remained statistically significant after controlling for socio-demographic and psychosocial factors. However, pointing in the same direction are the findings in the bivariate analyses, which suggest that also young age and not being married or in a stable relationship may contribute to increase the risk of being exposed to partner physical assault and/or sexual coercion. These assumptions do however need to be further investigated in larger samples.

### Methodological considerations

A major strength of this study is that the data comes from a randomised population-based sample of men and women in Sweden. To our knowledge, this was the first Swedish population-based study to explore both exposure and perpetration of IPV and its socio-demographic risk factors among men and women. Another strength in the current study is the use of the CTS2, a well-known violence instrument that is validated and used globally [17].

Among the limitations, the most notable is the rather low response rate and the internal drop- out rate related to the violence items, especially for men. Since the majority of external and internal drop-outs were unmarried, it might be that they had no experience of intimate relationships and therefore were not further motivated to answer the violence-related items. Further, those individuals most exposed and/or perpetrating violence might have been reluctant to fill in the questionnaire because of shame or fear of being identified, which has been found in an earlier study [5]. We believe also that this questionnaire may not be ideal for data collections through mailed questionnaires as the violence items are profuse and rather detailed in content. Another limitation is that the low number of respondents reduced the power of the analysis.

Since the data is based on self-reports of exposure and perpetration, care must be taken in comparing the results from this study with police reports or clinical records as only the most serious cases in terms of injuries or ill-health will be registered in such reports and not the kind of ‘every day’ violence that goes unnoticed by authorities.

Past year prevalence figures are considered to be more accurate than earlier in life figures due to a reduced recall bias. This is obvious in the current study, particularly for men, as earlier in life estimates are lower or equal to estimates related to past year exposure apart from SC exposure. This might be explained by higher recall bias in men being less threatened by the violence and abuse exercised by women than vice-versa.

Since this is a cross-sectional study, it is not possible to make definite statements about the direction of the causal relationships. Lack of social support, for example, can be a risk factor for IPV exposure, but might also be the result of prolonged IPV exposure.

## Conclusions

The findings in this study indicate that both men and women perpetrate- and are exposed to physical violence and control tactics in the past year, which points at gender symmetry but this has to be said with caution as the motives were not explored.

Male exposure to IPV, physical, sexual and controlling behaviour should be further explored in quantitative and qualitative studies in order to get accurate and repeated measurements, but also a better understanding of the characteristics of the violence. Equally important is to further investigate women’s use of violence, its motives and trigger factors. In depth understanding of women’s and men’s use of violence and control tactics may challenge existing gender theories that aim to explain why such behaviours occur. New theories may have to be formulated and not until then can preventive measures and interventions be developed.

## Competing interests

The authors declare that they have no competing interests.

## Authors’ contributions

GK designed the study and directed all steps in the data collection. SL carried out all statistical analyses with support from GK. SL drafted the manuscript, GK read and revised the text until a final version was in place, which was carefully read and accepted by both authors.

## Funding

This study was supported by a grant from the Swedish Research Council.

## Pre-publication history

The pre-publication history for this paper can be accessed here:

http://www.biomedcentral.com/1471-2458/12/945/prepub
